# Septal flip flap after endoscopic unilateral resection of sinonasal tumors: a multicenter experience of 133 patients

**DOI:** 10.1007/s00405-026-10436-0

**Published:** 2026-07-19

**Authors:** Alessandro Vinciguerra, Mario Turri-Zanoni, Marco Ferrari, Vittorio Rampinelli, Florian Chatelet, Davide Mattavelli, Alberto Schreiber, Stefano Taboni, Federico Russo, Piergiorgio Gaudioso, Benjamin Verillaud, Cesare Piazza, Paolo Castelnuovo, Piero Nicolai, Maurizio Bignami, Philippe Herman, Paolo Battaglia

**Affiliations:** 1 Division of Otorhinolaryngology, Department of Biotechnology and Life Sciences, University of Insubria, ASST Lariana, Como, Italy; 2 Section of Otorhinolaryngology – Head and Neck Surgery, Department of Neuroscience (DNS), University of Padova, Padova, Italy; 3 Unit of Otorhinolaryngology – Head and Neck Surgery, Azienda Ospedale- Università Padova, Padova, Italy; 4 Unit of Otorhinolaryngology-Head and Neck Surgery, ASST Spedali Civili Brescia, Department of Medical and Surgical Specialties, Radiological Sciences, and Public Health, University of Brescia, Brescia, Italy; 5 Otorhinolaryngology and Skull Base Center, AP-HP, Hospital Lariboisière, Université Paris Cité, Paris, France; 6 Unit of Otorhinolaryngology, Manerbio and Desenzano Hospitals, ASST del Garda, Brescia, Italy; 7 Division of Otorhinolaryngology, Department of Biotechnology and Life Sciences, University of Insubria, Ospedale di Circolo, Varese, Italy

## Abstract

**Purpose:**

Skull base reconstruction after endoscopic resection of sinonasal tumors remains challenging, particularly in preventing cerebrospinal fluid leaks. Autologous grafts are traditionally favored, whereas non-autologous materials may reduce donor-site morbidity with similar success. In this study, we explore surgical outcomes in patients undergoing unilateral resection of sinonasal tumors reconstructed with the septal flip flap (SFF), focusing on the impact of graft type on the efficacy and safety of the reconstructive strategy.

**Methods:**

This multicenter retrospective cohort study included adult patients with malignant sinonasal tumors treated via unilateral endoscopic resection and SFF reconstruction. Clinical and surgical variables were analyzed to identify predictors of early postoperative complications and factors influencing hospitalization using univariate and multivariate methods.

**Results:**

A total of 133 patients were included. Early postoperative complications occurred in 9.7%, with 4.5% related to skull base reconstruction. No variables, including graft type, significantly predicted SB-related complications in univariate logistic regression. Autologous grafts were associated with increased use of adjuvant radiotherapy (67.1% vs. 36.8%, *p* = 0.015). Conversely, the median hospitalization was 7 days. Dura mater resection, autologous graft use, early complications, and prior treatment were independent predictors of prolonged hospitalization at multivariate linear regression.

**Conclusion:**

This multicenter study confirms SFF as a reliable and safe option for anterior skull base reconstruction following unilateral sinonasal malignancy resection. The technique achieved robust closure, no flap necrosis, and low reconstruction-related complication rates. Graft type did not significantly influence outcomes, although autologous grafts provided better integration in patients receiving adjuvant radiotherapy, while non-autologous substitutes proved equally safe and effective in selected unimodal settings.

## Introduction

Cerebrospinal fluid (CSF) leak is one of the most frequent perioperative complications after transnasal endoscopic surgery to treat sinonasal malignancies, with an incidence of 0.5-6% of cases [1]. The presence of this adverse event can be precursory to more life-threatening complications (i.e. ascending bacterial meningitis, cerebral abscess, and pneumocephalus) which justifies the need for prompt and appropriate treatment [2]. Factors that have been described to mainly affect the occurrence of peri-operative CSF leak after transnasal endoscopic resection of the anterior skull base (SB) tumors are [1]: (1) the two-layers reconstruction of the defect, in contrast with the three-layers technique, known to be the gold standard procedure for large defect of the anterior SB; (2) application of non-autologous material, in contrast to the widely used autologous one, which may derive from fat, connective tissue, muscle, cartilage and bone. However, among all, the iliotibial tract (ITT) graft is the most used one due to its intrinsic characteristics of robust, elastic and versatile connective tissue that is easily vascularized after implantation and with excellent integration [2–4]. Furthermore, the application of autologous material results in a consequent external scar, increased surgical time, and potential complications [2, 5]. To address these limitations, recent studies have found, on the contrary, that non-autologous material, primarily porcine submucosa and collagen-based matrices, has a success rate for dural reconstruction that appears similar to ITT, but with reduced morbidity at the donor site and perioperative complications [6–8]. As a consequence, due to the conflicting data on the effectiveness and safety of non-autologous material, the application of these grafts in the reconstruction of large SB defects is not standardized and frequently underreported [6, 9, 10].

**Fig. 1:**
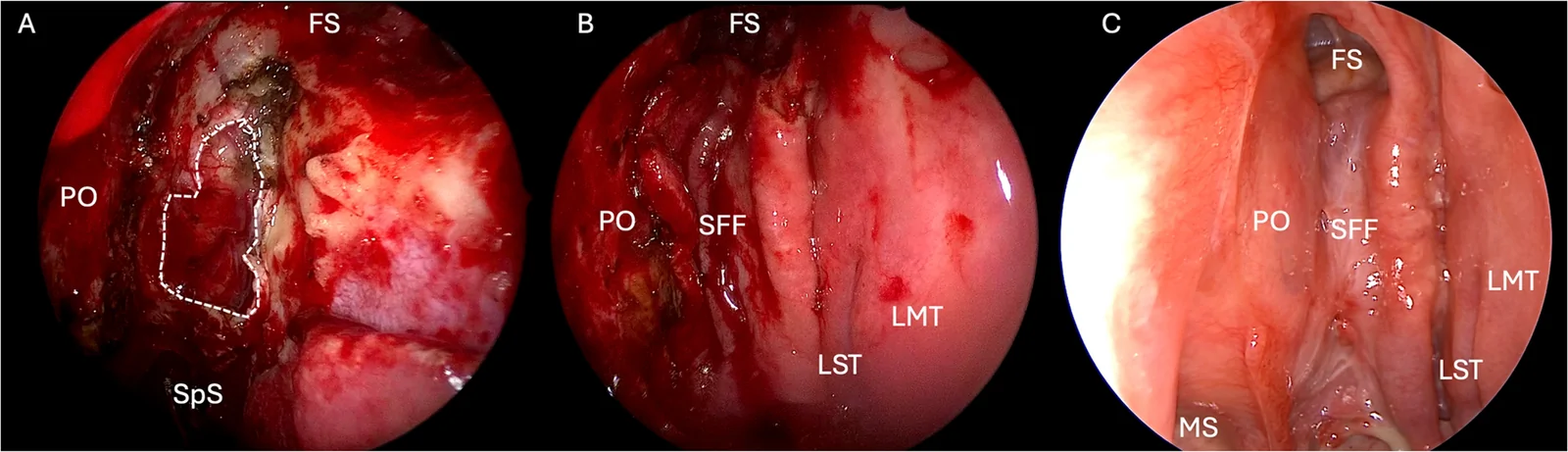
Endoscopic 45° images of a right anterior skull base reconstruction using SFF. (**A**) Intraoperative image of the resected dura mater of the right anterior skull base; (**B**) After having reconstructed the skull base with fascia lata, the SFF was rotated to resurface the skull base defect; (**C**) Post-operative endoscopic control two months after surgery FS= frontal sinus; LMT= left middle turbinate; LST= left superior turbinate; MS= maxillary sinus PO= periorbita; SFF= septal flip flap; SpS= sphenoidal sinus; white dotted line in figure A) indicates the boundaries of the anterior skull base defect

Another factor described that plays an important role in the prevention/management of perioperative CSF leak is the application of vascularized flaps, particularly the local ones [11]. However, in the oncologic scenario of sinonasal tumors, local flaps (i.e. nasoseptal flap, NSF) are rarely available so that regional pedicled flaps (temporo-parietal and pericranial flap) are frequently the main protagonist of vascularized SB reconstruction [12, 13]. Nevertheless, in selected cases in which the nasal septum and the contralateral nasal fossa are not involved, several local pedicled flaps can be used. Standard, posteriorly-pedicled NSF can be difficult to apply in the most anterior portion of an anterior SB defect, which variably involves the cribriform plate, ethmoidal roof, and posterior frontal plate. In recent years, our group has introduced the septal flip flap (SFF) pedicled on septal branches of ethmoidal arteries for the reconstruction of unilateral SB defect [14, 15]. This “new” reconstructive flap has been described as a valid solution in unilateral endoscopic craniectomies due to its solid vascular pedicle, high success rate, easy harvesting technique and low perioperative complication rate (Fig. 1) [15].

This manuscript aims to investigate predictors of surgical outcome following SB reconstruction in a cohort of patients treated with the SFF, with particular attention to the influence of graft type (autologous vs. non-autologous).

## Methods

This multicenter retrospective cohort study was conducted at five tertiary referral centres, with patients’ data retrieved from the MUSES database [1, 16, 17].

**Fig. 2:**
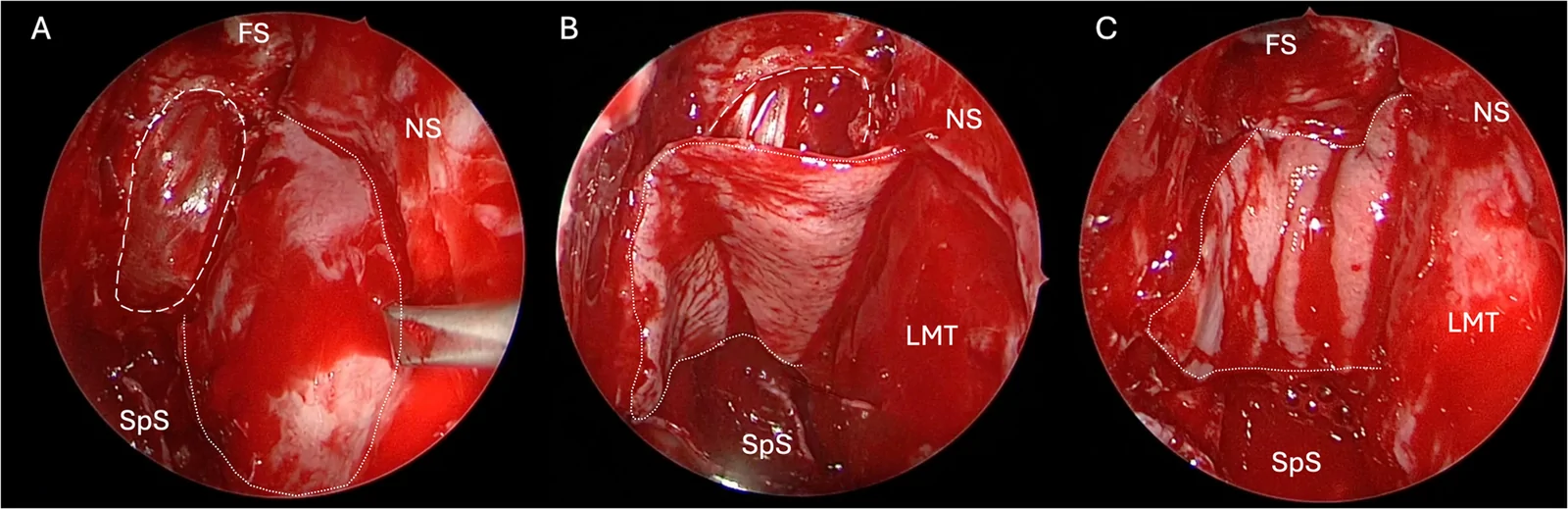
Endoscopic 0° images of the right nasal cavity following right endoscopic endonasal dural resection, demonstrating the step-by-step preparation of a septal flip flap. (**A**) The flap (dotted white line) must be harvested down to the floor of the nasal cavity and, where necessary, extended to incorporate the mucosa of the floor itself, to ensure adequate coverage of the reconstructed dural defect (dashed white line). (**B**) Once the incisions have been completed, the flap must be rotated over the dural defect to cover its entire extent (**C**). To maximise the reconstructive effect of the flap, its length should be sufficient to reach the lamina papyracea of the ipsilateral nasal cavity FS= frontal sinus, LMT= left middle turbinate, NS= nasal septum, SpS= sphenoid sinus

We included adult patients (≥ 18 years) with malignant sinonasal tumors treated with curative intent by unilateral transnasal endoscopic resection and reconstructed with the SFF, between January 2011 and December 2024. The SFF was considered suitable when there was no radiological or intraoperative evidence of tumor involvement of the cranial nasal septum, nor extension across the dura, or intradural space. The harvesting technique for the SFF followed the method described by Battaglia et al. (Fig. 2) [15].

Patients with benign lesions, open craniofacial resections, tumors involving the nasal septum (based on pre-operative radiology) or incomplete data were excluded. Clinical and oncologic data were collected from institutional databases including age, sex, laterality, tumor histology, TNM stage (AJCC 8th ed.), presentation (primary vs. recurrent-persistent), prior treatments, hospitalization duration, occurrence of complications and follow-up.

Surgical details included the extent of SB resection (mucosa, bone, dura), graft type for duraplasty (autologous vs. non-autologous), and the number of layers used for reconstruction. The removal of the crista galli was performed selectively, depending on the extent of bony and dural resection of the skull base. Autologous grafts were primarily harvested from the iliotibial tract (ITT) of the thigh; non-autologous options included extracellular matrix (ECM) based scaffold and collagen-based matrices. Graft selection was based on intraoperative considerations and surgeon preference.

Postoperative complications were categorised as early (occurring within 30 days) or late, graded according to the Clavien–Dindo classification [18]. All patients were followed with regular clinical and endoscopic evaluations, as previously described [1].

Informed consent was obtained from each patient for treatment and use of de-identified clinical data for study purposes; the study was conducted according to the ethical standards of the Declaration of Helsinki revised in 2011 and was approved by the institutional review boards of all participating centers (ASST Spedali Civili Brescia: protocol NP3616; University of Insubria: Insubria Board of Ethics, approval number 0033025/2015 (Ospedali di Circolo e Fondazione Macchi, Varese) and 212/2024 (ASST Lariana, Como); Hopital Lariboisie University of Paris: REFCOR database approval CNIL #91204 and CCTIR #11.337; Azienda Ospedale-Università Padova (“Comitato Etico Territoriale Area Centro-Est Veneto”): 433n/AO/23).

### Aims of the study

The primary aim of this study was to identify predictors of early postoperative complications in patients undergoing unilateral endoscopic resection of sinonasal malignancies assisted with SFF, with a specific focus on complications related to SB reconstruction and their association with the use of autologous versus non-autologous grafts for SB reconstruction. The secondary aim was to investigate clinical and surgical variables potentially influencing the length of postoperative hospitalization.

### Statistical analysis

Statistical analysis was conducted using SPSS (version 24, IBM Corp., Armonk, NY, USA). A normality test was applied to continuous variables, which were then treated accordingly. Non-normally distributed variables were analyzed with non-parametric tests. Categorical variables were analyzed using the Chi-square test. To identify predictors of early complications, a univariate logistic regression analysis was performed. Univariate linear regressions were conducted, after confirming the normal distribution of the residuals, to explore factors influencing hospitalization length. Variables with *p* < 0.05 were included in a multivariate linear regression model.

Results were expressed as beta coefficients or odds ratios with corresponding p-values.

A p-value < 0.05 was considered statistically significant.

## Results

A total of 133 patients were included in this study. Demographic, surgical, and oncological characteristics are summarized in Table 1. Most patients presented with primary tumor (77.4%, vs. 21.8% of persistent-recurrent), with intestinal-type adenocarcinoma (ITAC) being the most frequent histological subtype (52%), followed by olfactory neuroblastoma (ONB, 23.6%). In terms of tumor clinical T category, T2 (38.6%) and T3 (29.7%) were the most frequent.

### Complications

The overall complication rate was 15.7%; early post-operative complications occurred in 9.7% (13/133) of patients, while complications related to SB reconstruction were reported in 4.5% (6/133) (Tables 1 and 2). CSF leak after skull base reconstruction occurred in 2/107 (1.8%).

**Table 1:** Clinical, surgical and outcomes characteristic of patients involved in the study

Variable	Value (%), *N*°= 133
Clinical characteristics	
Age (years), median (IQ)	64 (55–72)
Sex, N (%)	
Female	36 (27.1)
Male	97 (72.9)
Side, N (%)	
Right	67 (49.6)
Left	66 (50.4)
Clinical presentation of the sinonasal tumor, N (%)	
Primary	103 (77.4)
Persistent-recurrent	30 (22.6)
Previous treatment	
None	103 (77.4)
Surgery	23 (17.3)
Surgery + RT	1 (0.8)
CHT	6 (4.5)
Pre-operative diagnosis, N (%)	
ITAC	66 (52)
ONB	30 (26.4)
NITAC	10 (7.2)
Mucosal melanoma	4 (3.1)
SCC	4 (3.1)
SNUC	4 (3.1)
Chondrosarcoma	4 (3.1)
ACC	3 (2.2)
Missing data	8
cTNM (8th edition), N (%)	
cT1	12 (11.9)
cT2	39 (38.6)
cT3	30 (29.7)
cT4a	13 (12.9)
cT4b	7 (6.9)
cN0	133 (100)
Surgical characteristics	
Dura mater resection, N (%)	
No	24 (18.8)
Yes	107 (81.7)
Missing data	2
Duraplasty graft, N (%)	
Autologous	79 (80.6)
Non autologous - Porcine small intestinal submucosa graft - Collagen-based dural graft matrix	19 (19.4) - 4 (21.1) - 15 (78.9)
Missing data	9
N° of layer of skull base reconstruction after dural resection (flap included), N (%)	
2	21 (19.4)
3	86 (80.6)
Margin status, N (%)	
R0	126 (95.5)
R1	6 (4.5)
Missing data	1
Adjuvant radiotherapy, N (%)	
No	48 (36.4)
Yes	84 (63.6)
Missing data	1
Outcomes	
Early post-operative complications, N (%)	
No	120 (90.3)
Yes	13 (9.7)
Early post-operative complications related to skull base reconstruction, N (%)	
No	127 (95.5)
Yes	6 (4.5)
Late complications, N (%)	
No	125 (87.2)
Yes	8 (6.0)
Treatment related death	0 (0)
Follow-up (months), median (IQ)	41 (13–65)

*RT* radiotherapy, *CHT* chemotherapy, *ITAC* intestinal type adenocarcinoma, *NITA* non-intestinal type adenocarcinoma, *ONB* olfactory neuroblastoma, *SCC* squamous cell carcinoma, *SNUC* , sinonasal undifferentiated carcinoma, *ACC* adenoid cystic carcinoma

**Table 2:** Postoperative complications classified according to the Clavien–Dindo classification

Clavien–Dindo classification	Event	*N*°
Grade 1	Pneumoencephalus	1
Grade 2	Delirium	1
	Meningitis	1
	Pneumonia	1
Grade 3a	ITT donor site complication	1
Grade 3b	Epistaxis	4
	CSF leak requiring surgery	1
	Cerebral abscess	1
Grade 4a	Stroke	1
	Posterior cerebral artery occlusion	1

Late complications occurred in 6% of cases, with four cases (3%) of frontal stenosis/mucocele, three middle ear effusions (requiring surgical drainage) and one nasolacrimal duct obstruction. No treatment-related or postoperative deaths were observed in the study cohort.

No complete or partial SFF necrosis was recorded throughout the study period.

When stratified by graft type, patients reconstructed with non-autologous materials experienced a higher rate of early complications related to SB reconstruction (2/19, 10.5%) compared to those with ITT autologous grafts (4/79, 5.1%). In only one case was the non-autologous graft associated with early post-operative meningitis, and it was treated with revision surgery and substitution of the non-autologous graft with an autologous one (ITT).

Despite these trends, univariate logistic regression analysis (Table 3) did not identify any significant predictors of early post-operative complications related to SB reconstruction. Neither age (*p* = 0.8), graft type (*p* = 0.55), presentation (*p* = 0.54), prior treatment (*p* = 0.63), nor the number of layers of SB reconstruction (*p* = 0.62) were associated with an increased risk.

**Table 3:** Univariate analysis of factors influencing the occurrence of early complications related to skull base reconstruction

Variable	Univariate OR (95% CI)	*p*-value
Age	0.99 (0.92–1.07)	0.800
Graft (autologous vs. non-autologous)	0.44 (0.03–6.63)	0.551
Presentation (primary vs. recurrent/persistent)	0.25 (0.003–21.97)	0.541
N° of layer of skull base reconstruction	1.85 (0.17–20.60)	0.619
Previous treatment	0.42 (0.01–15.04)	0.632

Among all cases, adjuvant radiotherapy was more frequently administered in patients reconstructed with autologous ITT grafts compared to non-autologous materials (67.1% vs. 36.8%, *p* = 0.015).

### Hospitalization

The median duration of hospitalisation was 7 days (range, 1–35 days).

When analyzing the variables that could have influenced the hospitalization time, the univariate linear regression analysis (Table 4) showed that dura mater resection (B = 2.93 days, *p* < 0.001), presence of early complications (B = 3.37 days, *p* = 0.016), SB reconstructed with autologous grafts (B = 1.98, *p* = 0.009) and patients with tumors treated previously (B = 0.87, *p* = 0.042) were associated with increase hospitalization time. At multivariate analysis, four variables confirmed as independent predictors of increased hospitalization duration: dura mater resection (B = 1.78, *p* = 0.010), autologous graft use (vs. non-autologous) (B = 2.03, *p* = 0.004), early complications (B = 3.27, *p* = 0.005), and previous treatment (B = 1.48, *p* = 0.013).

**Table 4:** Unstandardized coefficients (B) and p-values from univariate and multivariate linear regression analyses assessing predictors of hospitalization length (in days). Only variables with p < 0.05 in univariate analysis were included in the multivariate model. Statistically significant results are highlighted in bold

Variable	B (Univariate)	*p*-value (Univariate)	B (Multivariate)	*p*-value (Multivariate)
Age	-0.012	0.641	-	-
cT	0.606	0.122	-	-
Dura mater resection (yes vs. no)	2.928	**> 0.001**	1.778	**0.010**
Early complications (yes vs. no)	3.371	**0.016**	3.267	**0.005**
Graft (autologous vs. non-autologous)	1.976	**0.009**	2.031	**0.004**
Previous treatment (yes vs. no)	0.874	**0.042**	1.481	**0.013**

## Discussion

The main outcomes of the present study are: (1) the complication rate related to unilateral SB reconstruction with SFF was reported in 4.5% of cases with no predictors identified at logistic regression, underlying the similar outcome of graft used (autologous vs. non-autologous) in the context of a reconstruction assisted with SFF; (2) the reliability of SFF is excellent (no event of partial or complete necrosis); (3) the hospitalization time was influenced at multivariate analysis by several factors, namely previous treatment (e.g. radiotherapy, B = 1.48, *p* = 0.013), occurrence of early complications (B = 3.27, *p* = 0.005), dural resection (B = 1.78, *p* = 0.010) and the use of autologous graft (B = 2.03, *p* = 0.004). Nevertheless, adjuvant radiotherapy was more frequently administered in patients reconstructed with ITT graft (67.1% vs. 36.8%, *p* = 0.015), underlying that the patient’s selection is a crucial step in applying synthetic materials. To the best of our knowledge, this paper represents one of the largest series of SFF that not only validates this reconstruction solution clinically but also underlines the applicability of non-autologous graft in the reconstruction of large SB defects.

Sinonasal local flaps have been described as important protagonists in the surgical success of SB reconstruction; however, their use requires careful consideration in the context of sinonasal tumors not to infringe the oncologic principles of resection with clear margins [9]. Particularly, local flaps allow, compared to extra-nasal tissue placed overlay, a faster healing process with reduction of post-operative crusting and CSF-leak, through the resurfacing of the surgical field with autologous vascularized mucosa [15, 19]. Nevertheless, it should be noted that the application of local vascularized flaps represents a rarity in anterior SB tumors and, when feasible, it is the last step of SB reconstruction, which has to be watertight from the first layer, commonly represented by ITT [12]. Historically, the anterior cranial fossa reconstruction has been considered easier compared to other areas thanks to: (1) the gravity that pulls the frontal lobe on the inlay graft, though helping the material to be kept in position; (2) the favourable geometry, with a large lain plane helping the epidural dissection; (3) the lower CSF pressure, and the absence of large subarachnoid cisterns; (4) the presence of bony structures able to buttress the reconstruction. Unfortunately, when dealing with unilateral SB reconstruction, the presence of the falx cerebri, medially, prevents a complete epidural dissection so that the second layer, commonly in the epidural space, is placed overlay in its medial portion leading to a hybrid reconstruction compared to the classic SB reconstruction (first layer intradural, second layer overlay medially and in the epidural space anteriorly, posteriorly and laterally) [9, 15, 20].

Considering the above-mentioned criticisms and the difficulty in using the posteriorly-pedicled NSF to reach the far anterior border of anterior skull base defects, the SFF was introduced in 2016 and became one of the principal protagonists of vascularized reconstruction after unilateral SB resection [15]. Indeed, this flap ensures on one hand a reliable option thanks to its solid vascular pedicles (namely the anterior and posterior ethmoidal arteries), as confirmed by our series with no case of partial or complete flap necrosis; on the other hand, it guarantees a robust reconstruction thanks to its ideal arc of rotation to reconstruct the ethmoidal roof and seal efficiently the medial border of reconstruction, commonly considered as the weakest point [21]. Up to date, no solid evidence is available on the pros of SFF compared to the NSF, even if encouraging results, as sustained by the present paper, are emerging [22].

Regardless of the flap used, the process of wound healing after dura mater resection seems to occur by integration of the autologous material or, if a synthetic material is applied, through endogenous fibroblast migration underneath the flap applied, ultimately replacing the intra-cranial grafts and resulting in a thick scar [9]. The most used reconstructive material, before the ultimate flap application, is represented by the ITT thanks to its intrinsic characteristics and good integration of the autologous connective tissue. However, to harvest such material, a tight incision is required with consequent additional operative time and potential secondary morbidity. In particular, the most frequent immediate complications reported are hematoma (1%), seroma (3%), and about 40% of patients report mild tight symptoms (discomfort, mild pain or muscle prolapse) [5, 23–25]. To overcome these adverse events, non-autologous materials (artificial and biocompatible) have been introduced to reconstruct the dura mater defect: particularly, among all, the ECM-based scaffold and collagen-based matrices [26, 27]. These synthetic materials are characterised by an acellular skeleton composed of collagen, eventually associated with bioactive proteins and cytokines, that provide a framework for cellular integration of the host tissue, in a mean time of 30–60 days, while remaining relatively inert to immunogenic response [28, 29]. However, for some, their mechanical strength and liquid retention capacity may be lower compared to ITT and, especially in the early phases, their integration may be variable [26, 30].

**Fig. 3:**
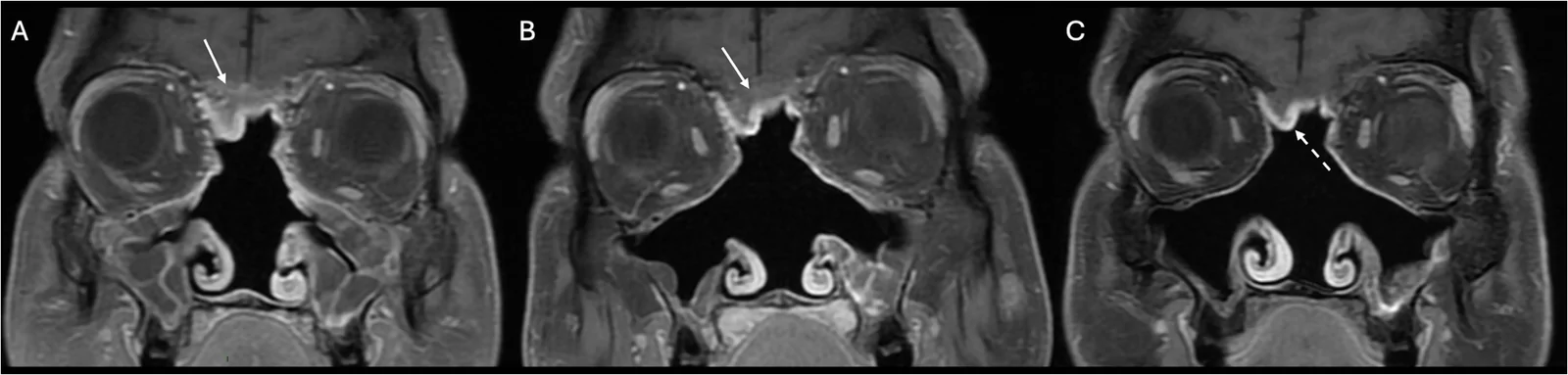
Contrast-enhanced coronal T1-weighted MRI showing intracranial inflammation, evidenced by contrast enhancement (white arrow), after 2 months of skull base reconstruction assisted with a xenogenic material (e.g. porcine submucosa matrix) (**A**), with progressive resolution at 6 months (**B**) and 12 months (**C**). Notably, contrast enhancement of the septal flip-flap persists over time (dotted white arrow), indicating stable vascular integration. It is important to note that the use of xenogenic grafts, such as porcine-derived materials, can elicit a local inflammatory response that may persist for up to 12 months, and should not be underestimated in clinical follow-up

**Fig. 4:**
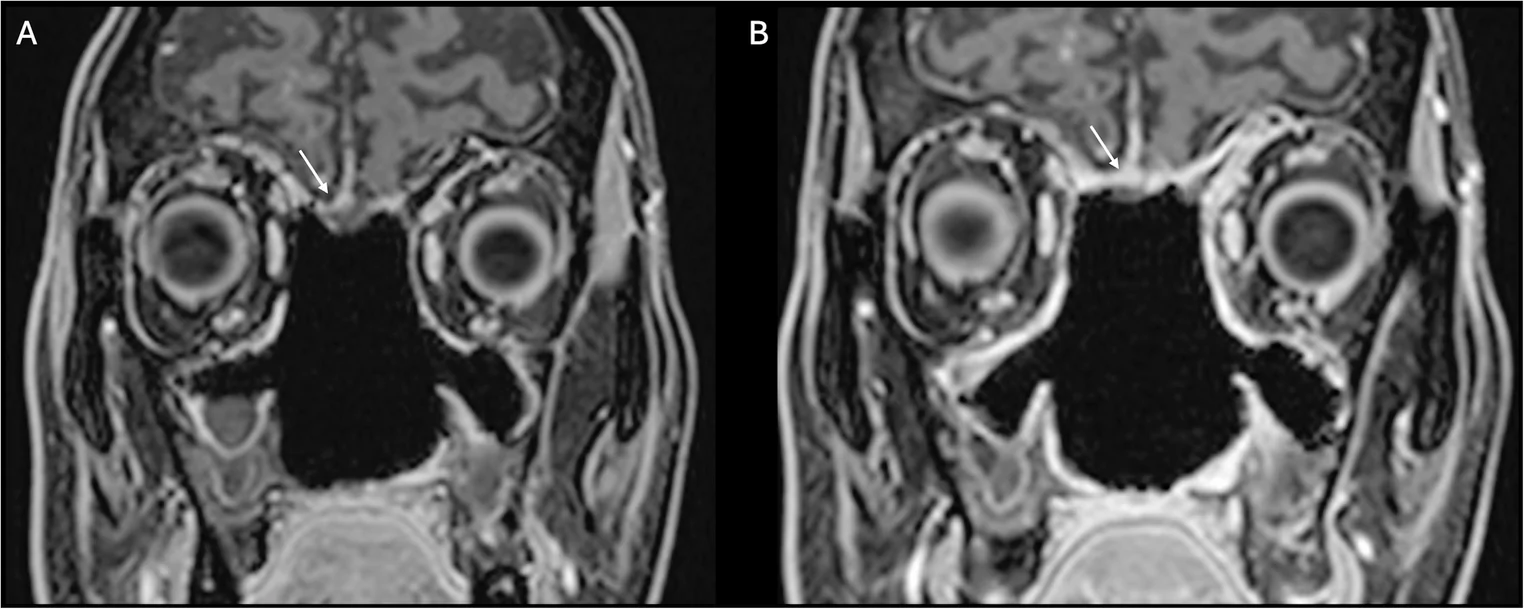
Contrast-enhanced coronal T1-weighted MRI images showing the radiological evolution following dural reconstruction with autologous material and application of a septal flip-flap after endoscopic endonasal right dural resection for an intestinal-type adenocarcinoma. In contrast to the use of xenogenic materials, both in the immediate postoperative period (**A**, 2 months postoperatively) and in the medium term (**B**, 8 months), the absence of intracranial contrast enhancement is evident, with the characteristic contrast uptake at the site of the pedicled flip-flap (indicated by the white arrow) which, in this case, is more pronounced at 8 months

A recent meta-analysis of 29 studies demonstrated that there is no significant difference in the occurrence of post-operative CSF leak or meningitis between autologous and non-autologous SB reconstruction, reinforcing the role of these synthetic materials [31]. However, in the data analysis of the latter paper, no stratification was performed based on whether or not a local flap was applied, leaving uncertainty regarding the true clinical effectiveness of these non-autologous grafts. Indeed, many authors have underlined the necessity of non-autologous graft coverage to avoid extrusion and superinfection, even if non-conclusive evidence is available to date [6, 8, 32]. In our experience of SB reconstruction assisted with a pedicled SFF, the graft type applied (*p* = 0.55), together with age (*p* = 0.8), presentation (*p* = 0.54), prior treatment (*p* = 0.63) and number of layers of SB reconstruction (*p* = 0.62) were not predictors of early post-operative complications. Nevertheless, it should be noted that, from a descriptive point of view: (1) non-autologous materials experienced a higher rate of early complications (2/19, 10.5%) compared to those with ITT (4/79, 5.1%); (2) adjuvant radiotherapy was more frequently administered in patients reconstructed with autologous ITT grafts (*p* = 0.015). Indeed, synthetic materials seem to generate thick and resistant scars which is entirely based on fibroblast cell migration, a slower process compared to the integration and revascularization of autologous grafts. This difference may favour ITT in bimodal settings (surgery associated with RT), as it provides a more robust and faster reconstruction that, in addition, remains reliable also when flap coverage is suboptimal (e.g., very anterior or posterior resections), whereas non-autologous grafts are more vulnerable to infection or dissolution when exposed. However, since most patients undergoing SFF-based reconstruction are candidates for unimodal treatment, the clinical impact of this biological distinction remains limited. An aspect that is sometimes underestimated is that, given the pathophysiological principles underlying the application of synthetic dural substitutes, an intradural inflammatory reaction in the immediate postoperative period is a physiologically constant finding, which in some cases may be mistaken for early intracranial tumour recurrence (Fig. 3). This type of radiological finding is not observed when an autologous dural substitute is employed (Fig. 4).

Moreover, our analysis on hospitalization time underlines that autologous graft use (vs. non-autologous) (B = 2.03, *p* = 0.004), together with early complication (B = 3.27, *p* = 0.005), dural resection performed (B = 1.78, *p* = 0.010) and previous treatment (B = 1.48, *p* = 0.013), predicted a longer hospital stay. Although it remains unclear how the application of an autologous graft may influence the length of hospitalisation, likely due to donor site morbidity, this data underlines the potential benefit of synthetic materials when applied in selected cases and covered with a vascularised flap [9].

The SFF coverage after unilateral dura mater resection has an indirect additional advantage: this flap indirectly covers the posterior border of the enlarged frontal sinusotomy, necessary to complete a proper SB approach, so that it theoretically decreases post-operative stenosis of the frontal sinus. Our series describes this adverse event in 4.5% (6/133) of cases, in contrast with the 20% reported in a recent meta-analysis of enlarged frontal sinusotomies without mucosa resurfacing [33]. This data is a rarity in the literature considering that mucosa resurface in the context of sinonasal malignant resection is unusual and under-reported; however, if the SB reconstruction is performed with the SFF, indirectly the flap will cover the posterior border of the frontal sinusotomy, thus increasing the functional outcome.

This study presents some limitations: firstly, its retrospective nature, which may introduce selection and information biases. Moreover, despite the multicentric design, the relatively limited sample size in the non-autologous graft group may have affected the statistical power to detect subtle differences in outcomes. Thirdly, graft selection (autologous vs. non-autologous) was not standardized by uniform criteria, but rather left to the surgeon’s intraoperative preferences, thou representing a potential source of bias.

## Conclusions

This multicenter study confirms the SFF as a reliable and safe option for anterior skull base reconstruction after unilateral resection of sinonasal malignancies, with no flap necrosis and a low rate of reconstruction-related complications. The SFF ensured robust closure and may also reduce the risk of frontal sinus stenosis, supporting its role as the cornerstone of this reconstructive strategy. Within this framework, graft type did not significantly affect complication rates, but autologous grafts were associated with longer hospitalization while providing a more reliable integration in patients requiring adjuvant radiotherapy. Conversely, non-autologous substitutes, when covered by a vascularized flap such as the SFF, proved similarly safe and may represent a valuable alternative in selected unimodal settings.
